# A Review on Bubble Stability in Fresh Concrete: Mechanisms and Main Factors

**DOI:** 10.3390/ma13081820

**Published:** 2020-04-12

**Authors:** Xiaohui Zeng, Xuli Lan, Huasheng Zhu, Haichuan Liu, Hussaini Abdullahi Umar, Youjun Xie, Guangcheng Long, Cong Ma

**Affiliations:** 1 Department of Civil Engineering, Central South University, Changsha 410075, China; 2 Department of Civil Engineering, Ahmadu Bello University, Zaria 810107, Nigeria

**Keywords:** bubble stability in concrete, film strength, gas diffusion rate, surface tension, nano-silica

## Abstract

In order to improve the stability of air bubbles in fresh concrete, it is of great significance to have a better understanding of the mechanisms and main influencing factors of bubble stability. In the present review, the formation and collapse process of air bubbles in fresh concrete are essentially detailed; and the advances of major influencing factors of bubble stability are summarized. The results show that the surface tension of air–liquid interface exerts a huge impact on bubble stability by reducing surface free energy and Plateau drainage, as well as increasing the Gibbs surface elasticity. However, surface tension may not be the only determinant of bubble stability. Both the strength of bubble film and the diffusion rate of air through the membrane may also dominate bubble stability. The application of nano-silica is a current trend and plays a key role in ameliorating bubble stability. The foam stability could be increased by 6 times when the mass fraction of nano-particle reached 1.5%.

## 1. Introduction

Concrete’s freeze-thaw damage [1] leads to premature cracking and other deterioration of concrete [2], which have seriously impaired the service life of concrete, and is one of the primary problems that affect the durability of concrete [3,4,5,6,7,8]. Air-entraining technology [9] is one of the most widely adopted technical approaches to improve the frost resistance of concrete and ameliorate durability problems [2,10,11]. Most of the concrete in North America, Northern Europe, Japan and other countries have adopted air-entraining technology. Some major projects in China (such as the Three Gorges Project [12], Qinghai–Tibet Line Project [13], etc.) have also adopted air-entraining technology.

**Figure 1:**
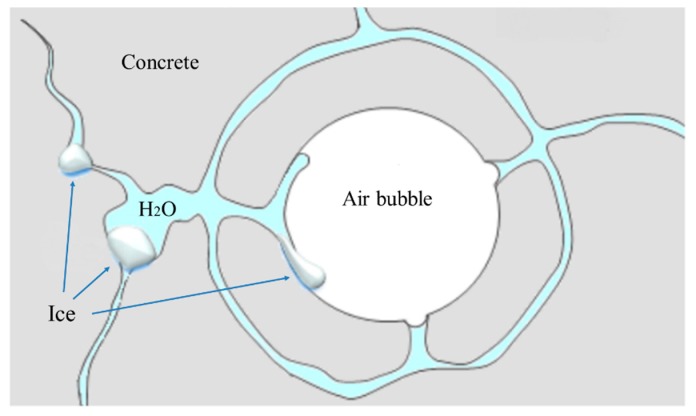
Basic principle of antifreeze of concrete with reasonable air bubble content.

Hardened concrete contains different categories of air voids [1,14,15] which are changing in volume during the hardening process [16]. A large quantity of tiny and stable air bubbles created by air-entraining agents act as “pressure-relief reservoirs” in hardened concrete [17,18,19]. So that when excess water is frozen, the ice expands and moves into the air voids, thus concrete damage is avoided [20]. The basic principle of antifreeze of concrete with reasonable air bubble content is depicted in Figure 1. Air-entrainment also improves workability and reduces the segregation of fresh concrete [21,22].

Air bubbles must remain stable so that they could be preserved in the fresh concrete and maintain a certain amount in solid concrete [23]. It has been reported that assuring appropriate number, spacing, and distribution of tiny entrained air bubbles in concrete can significantly affect the mechanical properties of concrete [24,25,26,27,28] and is critical for solving the destruction of freeze-thaw cycles [14,29,30]. This is because the distance the freezing water travels to the nearest air void is shortened [31]. These parameters (e.g., air void distribution, spacing, total and micro air void occurrence, and specific surface) in cementitious materials could be analyzed by micro X-ray computed tomography [32].

Since fresh concrete is a heterogeneous mixture with different phases, every constituent of the system affects the stability of air bubbles, and the corresponding mechanisms involved are complex. Generally, the factors affecting air bubble’s stability in concrete include the properties of basic concrete components [33,34], the mix proportion of concrete [35,36,37], the mixing process [33,38], temperature [39], atmospheric pressure [20,40], air-entraining agents [41,42,43], other chemical additives [44,45,46], mineral admixtures [47,48,49,50], and the quality of mixing water [15]. Each constituent of the system, either internal or external, influences bubble stability to a varying extent. Previous studies [15,51,52,53,54,55] contributed a lot to comprehensively introduce the influence of these factors on the stability of air bubbles in the concrete, but there was a lack of deep elaboration of the physical and chemical mechanisms.

In this paper, the formation and collapse of bubbles in concrete are characterized in an easy understand way; the major influencing factors of bubble stability containing surface tension of air–liquid interface, the viscosity of fresh concrete, the surface charge of the liquid film, the strength of the liquid film, the diffusion rate of gas through liquid film, air-entraining agents, superplasticizers, salt admixtures, mixing process, transportation, temperature, and atmospheric pressure, etc. are summarized on the basis of literature accessible, their mechanisms are expounded as well.

Although some factors such as the quality of mixing water, the mineral admixtures, and the properties of aggregates, etc. have not been detailed in the work, they are equally as important as the factors reviewed in the paper. For instance, the decrease of the maximum size of aggregate from 20 mm to 14 mm resulted in 1.7% higher air content [56].

The technology of nano-silica stabilized bubbles is presented and discussed to improve the stability of air bubbles in concrete.

## 2. Formation and Collapse of Air Bubbles in Concrete

### 2.1. Formation of Bubbles in Concrete

Fresh concrete consists of innumerable air bubbles generated during the mixing process of concrete [57,58,59], and a small amount of bubbles originated from the air dissolved in water. The bubble is composed of air entrapped inside and a thin external liquid membrane packing. The properties of the membrane are affected by the categories and arrangements of surfactants absorbed on it [60]. The membrane, which has micropores containing water, is the air–liquid interface of bubble and is one of the important factors affecting interphase mass transfer [61].

The formation of air bubbles in fresh concrete is closely related to the stirring process which not only causes the liquid to produce vortex and take in air, but also makes the larger bubbles in fresh concrete to be squeezed and crushed into small bubbles by aggregates. In addition, the process of stirring changes the pressure suffered by fresh concrete, which makes the solubility of air in water different, so the air is either released or absorbed. Powers [62,63] pointed out that the air bubbles were mainly entrained by the vortex which is generated during the stirring process, and entrapped by the three-dimensional barrier formed via the fine aggregates which were colliding with each other.

The formation of a bubble in fresh concrete requires [64] that the activation energy of vortex produced by stirring on the surface of fresh concrete must be greater than the surface tension of mortar, then the surface of mortar is deformed unevenly and a gap is generated, so that air is drawn into the fresh concrete to form bubbles [33,65,66]; the kinetic energy of fluid near the mortar surface must be enough to overcome the buoyancy to bring the formed bubbles deep into the interior of fresh concrete [67]. Reducing the surface tension of fresh concrete and increasing the rotation speed are both beneficial to the introduction of bubbles, and high-speed stirring can form small bubbles, which is conducive to the stability of air bubbles in fresh concrete [67].

### 2.2. Collapse of Bubbles in Concrete

According to thermodynamic law, air bubbles in fresh concrete are inherently unstable. Because there is surface free energy between the interface of the air bubble and fresh concrete. The surface free energy always tends to decrease and approach zero, macroscopically performing as the break-up of bubbles [68]. The deformation of the bubble is a prelude of break-up and is an indicator of destabilizing force, when the force is sufficiently big, the rapture of bubbles will occur [69]. Generally, the coalescence of bubbles is an important step of bubble collapse, and often induces break-up [70].

Myers [71] summarized the fundamental physical processes of bubble collapse, and pointed out that small bubbles became smaller due to the gas diffusion, or dissolved in a large amount of solution; the liquid film of adjacent bubbles ruptured, thus bubbles merged as a result of capillary flow; and the rapid drainage of liquid film between bubbles caused a burst of bubbles. Du and Kevin [15] believed that the major forms of bubble collapse in fresh concrete are the former two processes concluded by Myers.

#### 2.2.1. Gas Diffusion

Bubbles are in different sizes since they were created, and the pressure in small bubbles is higher than that in large bubbles. Mielenz et al. [58] suggested that the gradient of air pressure between small and large bubbles caused gas diffusion.

The diffusion rate of gas q [72] between two bubbles of radius R_1_ and R_2_ is given by Equation (1):

(1) q=−JAΔp,

where J is the permeability of diffusion path; A is the effective projected area of the interface position where diffusion occurs between the two bubbles, γ is the surface tension of the liquid.

The pressure difference Δp between two bubbles is given by Equation (2):

(2) Δp=2γ(1R1−1R2).

**Figure 2:**
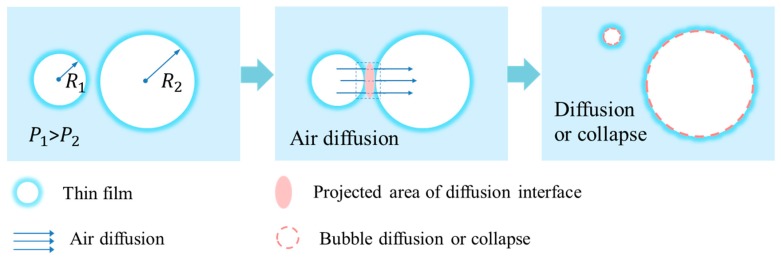
Gas diffusion between small bubbles and big ones.

The transfer of gas has changed the air content of both small and large bubbles. Since gas diffusion proceeds in the direction of lower pressure, small bubbles diffuse towards large ones through liquid film. Ley et al. [73] found out that the small bubbles became much smaller, while the large bubbles inflated after gas diffusion. As a result, small bubbles disappear and large bubbles collapse. The illustration of gas diffusion is demonstrated in Figure 2.

#### 2.2.2. Drainage of Liquid Film

**Figure 3:**
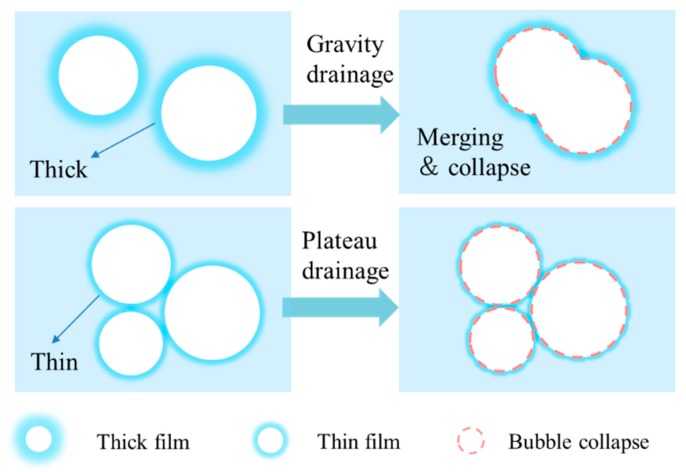
Gravity drainage and plateau drainage of bubble film.

When a fresh bubble is generated, the liquid film is thick, the drainage of the bubble’s liquid membrane is controlled by gravity drainage [74]. However, with the liquid film getting thinner and thinner, the drainage of bubble’s liquid membrane is dominated by plateau drainage. These two kinds of bubble drainage are shown in Figure 3.

The viscosity of cement paste plays a vital role in gravity drainage. Electrolytes or polymers that can increase the viscosity of the fresh concrete phase will reduce the drain rate of the liquid film. Surfactants can form viscous liquid crystals in bulk solution which helps to suppress the drainage effect, so that the stability of bubbles will be improved.

**Figure 4:**
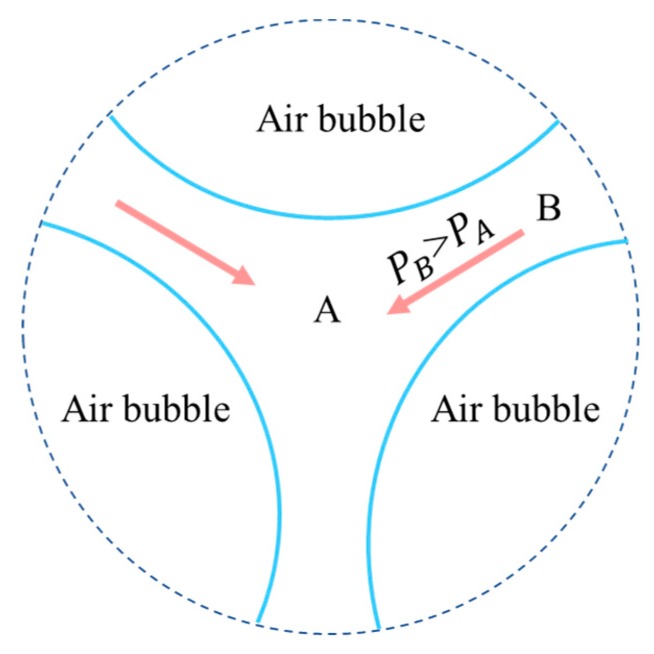
Principle of plateau drainage.

Plateau drainage is caused by the curvature difference of liquid film surface. The intersection of three or more bubbles will form Plateau boundary [72,75], as shown in Figure 4 [68]. The curvature of liquid film at the Plateau boundary is greater than that at the adjacent boundary formed by only two bubbles. The pressure of point B is higher than that of point A. It causes the liquid to flow from point B (outside the Plateau boundary) to point A (inside the Plateau boundary). The pressure difference of point A and point B is given by Equation (3) [72]:

(3) Δp=γ(1RB+1RA),

where R_A_ and R_B_ are the curvature radius of liquid film at point A and point B respectively; γ is the surface tension.

The liquid film becomes thinner because of Plateau drainage. When the bubble film is thin to some extent, bubble breaks [76]. The dynamic properties of the film are crucial for the drainage of bubble film [77]. The major factors influencing Plateau drainage include the surface tension of the liquid film and the radius of curvature at point A and point B. The greater the surface tension of the liquid film, the greater the pressure difference which leads to drainage, and the more easily the liquid film ruptures.

## 3. Factors Affecting the Stability of Air Bubbles in Fresh Concrete

Bubble stability is equally important to bubble generation. The basic properties of fresh concrete and bubbles directly affect the formation and stability of air bubbles, especially the surface tension which has been regarded as the most principal factor influencing bubble stability. To some extent, the significance of surface tension may be overestimated in view of the latest research. This section will show that the bubble stability is dominated by other factors as well as surface tension.

### 3.1. Properties of Fresh Concrete

#### 3.1.1. Surface Tension

**Figure 5:**
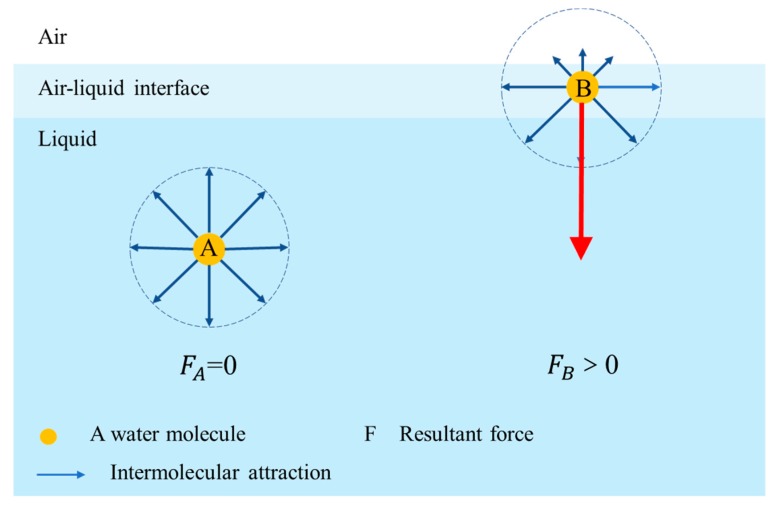
The resultant forces of molecules deep in water and on the surface.

Surface tension is one of the essential factors affecting the stability of bubbles [75,78,79]. Surface tension exerts its effect in the thin interfacial region between air and liquid [80], and the surface tension of liquid originates from the unbalanced forces on the molecules of liquid surface layer [81]. The forces on the molecules inside liquid can offset each other, so the molecules inside the liquid are at a state of force equilibrium; however, the attractive force from bulk molecules is much bigger than that of gas- phase molecules, because the density of the gas phase is lower, so that the molecules of the liquid surface layer are exposed to unbalanced forces which tends to pull the molecules of surface layer deep into the liquid, and the resultant force points to the liquid lower surface, as demonstrated in Figure 5 ( ‘A’ represents a molecule deep into the liquid, and the resultant force ‘F_A_’ is equal to ‘zero’ which means a balanced force, while ‘B’ represents a molecule on the surface of the liquid and the resultant force ‘F_B_’ is more than ‘zero’ which indicates an unbalanced force). The molecules of liquid surface layer suffer an unbalanced force, which macroscopically manifests as ‘surface tension’.

The formation of bubbles in fresh concrete will generate more gas–liquid interface thereby the surface free energy of the system increases. Reducing surface tension can directly decrease the free energy required for the generation of bubbles, which is beneficial for the formation of bubbles. Furthermore, surface tension affects the Plateau drainage process [82]. When surface tension lowers down, the hydraulic pressure difference between the Plateau boundary and other parts of the liquid film decreases. So that the draining speed of liquid film slows down, which is advantageous to the stability of air bubbles.

Surface tension not only affects the generation of air bubbles in fresh concrete, but also affects the drainage process of liquid film, thereby affecting the stability of air bubbles. Although reducing surface tension is favorable for bubble stabilization, it may not be the only determining factor. Miller et al. [83] Studied the relationship between the stability of the bubble’s liquid film and surface rheology, and believed that the bubble stability cannot be merely determined by surface tension. Systems with equal surface forces can have totally different stabilities. Rosen et al. [72] and Krause et al. [84] investigated the relationship between structures of surfactants and foaming properties, and concluded that surface tension is closely related to the foaming ability, but there was no one-to-one correspondence with the stability of bubbles. It was also suggested that bubble stability is controlled by other factors as well as surface tension. Only when the liquid film is strong enough to hold up bubbles, reducing surface tension can help to stabilize bubbles [75].

#### 3.1.2. Viscosity of Fresh Concrete

The rheological properties [56,85] of fresh concrete affect the system of air-voids [86], as well as the quality of hardened cementitious materials [87]. The behavior of fresh concrete can be considered as Bingham and represented by the Equation (4) [88,89,90]:

(4) $$\tau =\tau_{0}+\eta \dot{\gamma },$$

where τ is the shear stress (Pa), $\tau_{0}$ is the yield stress (Pa), η is the viscosity (Pa s), and $\dot{\gamma }$ is the shear rate (s^−1^).

Powers [62] pointed out that the shear yield stress of cement could prevent small bubbles escaping from the paste, and the buoyancy of small bubbles was not enough to break the surface of fresh concrete and go upwards. According to the Stokes’ law and the principle of buoyancy [91], when a bubble is floating in the fresh concrete, it is subjected to a resultant force of gravity, buoyancy, and viscous resistance caused by the movement between bubbles and the fresh concrete.

Both the viscosity of fresh concrete and bubble diameter determine the rising rate of the bubble. Decreasing the bubble diameter and increasing the viscosity of fresh concrete can reduce the floating speed of bubbles in fresh concrete, thereby reducing the collapse probability of bubbles floating to the surface of fresh concrete. In addition, high-viscosity of fresh concrete can prevent bubbles from rupturing when they are exposed to vibration or disturbance [75]. However, the high viscosity of fresh concrete is detrimental to the generation and stability of air bubbles [72].

The viscosity of fresh concrete is directly affected by water to cement ratio. When water to cement ratio is low, the viscosity of fresh concrete is high. To some extent, a high viscosity impedes the bubble’s rupture and coalescence by providing a “cushion effect” for air bubbles to keep bubbles free from external disturbances [92,93]. However, at the same time, the generation of bubbles is difficult; when water to cement ratio is high, the viscosity is low, so that bubbles merge and escape easily [35,36,55].

The viscosity of fresh concrete is reflected by the slump of fresh concrete. It is hard to entrain air bubbles if the slump is too small; it is easy to lose air bubbles if the slump is high; only when the slump is moderate, is it suitable for the formation and stability of bubbles. As a result, the gas content of fresh concrete is higher [37]. In accordance with these experimental results, the gas content of concrete decreases with the increasing of slump within a certain range, and the trend of dropping increases [34].

### 3.2. Bubble Properties

#### 3.2.1. Surface Charge of Liquid Film

It is generally believed that the strength of the liquid film and the separation pressure are important factors affecting the stability of bubbles [94]. Separation pressure is the electrostatic repulsion between the two sides of the liquid film, which prevents the thinning of the liquid film. Its essence are the intermolecular forces, which are mainly composed of van der Waals forces, electrostatic repulsion and short-range repulsion [95,96]. Carey and Stubenrauch [97] found through experiments that ionic active agents can increase the electrostatic repulsion in separation pressure. When the liquid film becomes very thin, the stability of the bubble relies on the electrostatic repulsion between the ion-adsorbing layers on both sides of the liquid film [72].

#### 3.2.2. Strength of Liquid Film

The strength of liquid film is one of the critical factors determining the stability of bubbles, as shown in Table 1. A membrane balance was used to study the strength of the monomolecular film of the air-entraining agent at the gas–liquid interface [98]. The strength of the monomolecular film determines the stability of bubbles entrained by the air-entraining agent. The higher the membrane strength, the more stable the air entrained bubbles, and the smaller the loss of gas content in concrete over time. The strength of the bubble film comprises the surface viscosity and surface elasticity of adsorption film on the surface of the liquid film [44,74,94].

The surface viscosity refers to the viscosity within a monolayer on the surface of a liquid film. This viscosity is caused by the interaction and the hydration of surfactant molecules between the hydrophilic groups in the surface monolayer. Surface viscosity is a decisive factor affecting the drainage process of liquid film. When the surface viscosity increases, the drainage rate of the liquid film slows down significantly. However, if the surface viscosity is too high or too low, it will not contribute to bubble stabilization [72]. With the increase of surfactant concentration, the flow velocity of liquid film surface decreases and the liquid film becomes stronger, so the stability of liquid film is enhanced [99]. The surface viscosity affects the balance of shear stress at gas–liquid interface [100].

The surface viscosity includes surface shear viscosity and surface expansion viscosity which is also referred to as expanded viscosity [101,102]. Some studies have discussed the influence of surface viscosity on the properties of liquid film interface through theoretical simulations and experimental researches. Matar [100] studied the stability of liquid film by linear stability theory and found that increasing the surface viscosity can delay the crack of liquid film. Lope [103] introduced a nonlinear equation in which surface viscosity was affected by the concentration of surfactant, and found out that the balance of radial stress was dominated by Marangoni stress, while the tangential stress was only related to the surface shear viscosity. Naire S et al. [104,105,106,107] studied surface viscosity and the evolution of surface viscosity with the concentration of surfactant, and pointed out that the surface viscosity had a stabilizing effect on the spreading disturbance.

Surface viscosity is a crucial factor affecting the drainage process of the liquid film, and with the increase of surface viscosity, the drainage rate of the liquid film slows down significantly.

The surface elasticity of bubble liquid film is also one of the decisive factors affecting the drainage process of liquid membrane. Surface elasticity refers to the special resilience when the liquid film is locally stretched or thinned by external forces. The special resilience arises from the migration of film-forming materials [72]. Saulnier et al. [108] and Sett et al. [109] concluded through experiments that liquid membranes with high surface elasticity had a longer lifetime, while liquid membranes with low surface elasticity had a shorter lifetime. Zang et al. [110] found that when silicon dioxide nano-particles had a contact angle at the gas–liquid interface of 90°, the surface elasticity of liquid film, the foaming property and the stability of foam reached the maximum. Furthermore, the relationship between the elasticity of surface film and the foam stability was studied [75]. The results showed that surface tension was not the single determining factor of foaming performance and foam stability. In the experimental system, the stability of the foam film was directly related to the surface elasticity. The systems with higher surface elasticity possessed higher foam stability, and the adding of polymers enables the surface viscosity of liquid film to increase, thereby inhibiting the liquid film from becoming thinner [75]. When the strength of the membrane is strong enough to form a bubble, lowering surface tension will help the bubbles to stabilize. The summary of previous research completed on bubble stability and the strength of the liquid film is shown in Table 1.

#### 3.2.3. Diffusion Rate of Gas through Liquid Film

Another factor that determines bubble stability is the gas diffusion rate through the liquid film from one bubble to another. The rate of bubble diffusion depends on the impediment to gas migration between air–liquid interfaces and the liquid between them, that is, the ability of gas to penetrate the liquid film.

Research suggested that the gas diffusion occurred in water-containing pores between surfactant molecules in the surface film [60]. The more closely arranged the molecules on the film surface, the more difficult for gas to penetrate through the liquid film, and the better the bubble stability [111]. Therefore, the close arrangement of surfactant molecules in the adsorption membrane was expected to reduce the gas diffusion rate between two bubbles. As the number of carbon atoms in the hydrophobic chain of surfactant molecule increasing and the molecular weight of the hydrophilic group decreasing, the barrier effect of interface on gas diffusion rises [61].

### 3.3. Admixtures

#### 3.3.1. Air-Entraining Agent

Air entraining agent (AEA) [9,10] is a kind of surfactant whose molecular structure is amphiphilic: one end is a hydrophilic group and the other end is a hydrophobic group [78]. Air-entraining agents affect the viscosity and bleed behavior of cement paste. The retention of air bubbles generates bridges between cement particles, resulting in an increase in viscosity and a decrease in bleeding of cement paste [20]. Additionally, air-entraining agents can remarkably increase both the number and content of air voids covering the whole diameter ranges, as well as change the system of air voids into finer size distributions, which plays a significant role in improving the durability of hardened concrete [14]. The reasons involved are explained as follows:

The use of air-entraining agents can greatly cut down surface free energy of the system by reducing the surface tension of the liquid. The formation of bubbles in cement paste increases the surface free energy of the system, so that the system is a thermodynamically unstable system [75]. The air-entraining agent can be directionally adsorbed at the gas–liquid interface, with the hydrophilic group facing the water phase and the hydrophobic group turning towards the gas phase. The original gas–liquid interface will be replaced by a new interface [72]. That is, the interaction of the original gas–liquid interface will be converted into the interaction between air-entraining agent molecule’s hydrophilic group and water molecules, as well as the interaction between air-entraining agent molecule’s hydrophobic group and gas-phase molecules. Because these interactions are much stronger than the interaction between gas-phase molecules and water molecules, the tension on both sides of the gas–liquid interface is decreased as a result of the presence of air-entraining agent molecules. The rising of free energy caused by the increase of surface area during the formation of bubbles in fresh concrete is reduced [72]. That is to say, the energy required for bubble formation is reduced, which contributes a lot to the formation and stability of air bubbles.

The strength of bubble film can be improved by air-entraining agents. Usually, the surfactant molecules could limit the thinning of bubble film and enhance film strength [78]. The molecules of air-entraining agent align on the bubble film to form a monomolecular elastic membrane, which enhances the strength and anti-extrusion ability of liquid membrane, reduces the possibility of bubble polymerization, enables the liquid film to become stronger and not collapse easily, and keeps numerous evenly distributed tiny bubbles stay stable eventually [1]. As for ionic air-entraining agent, the upper and lower surfaces of bubble liquid film are charged, thus forming a double electric layer structure. The electrostatic repulsion prevents the thinning of the liquid film and helps ameliorate bubble stability.

Furthermore, air-entraining agents enable the bubble film “self-healing”. When a bubble is locally thinned by external force and disturbance, the concentration of air-entraining agent molecules in the liquid film will decrease, the surface tension *γ* will increase [78]. So that a gradient of surface tension will be formed with other parts of the liquid film. Driven by the surface tension gradient, the liquid carrying air-entraining agent molecules will go back to the liquid surface thinned along the optimal route for repairing to maintain the dynamic balance of active molecules on the surface of bubble liquid film [72], so that the thickness, strength and surface tension of liquid film will recover. This phenomenon is called Gibbs surface elasticity. However, it takes time for surfactant molecules to diffuse to the thinned liquid film and restore the surface tension of the thinned film. This is the Marangoni effect. The Gibbs-Marangoni effect helps bubbles to maintain stability [112].

Many air-entraining agents stabilize bubbles by reducing surface tension, while some other AEAs depend more on calcium interaction [113]. These air-entraining agents could react with the cement clinker and calcium hydroxide, and generate insoluble sediment which is adsorbed on the bubble film, forming a solid shell. This shell improves the stability of air bubble, thereby extending its lifetime [94]. Many studies have observed this special shell through a scanning electron microscope (SEM), low-temperature scanning electron microscope (LTSEM) and environmental scanning electron microscope (ESEM).

**Figure 6:**
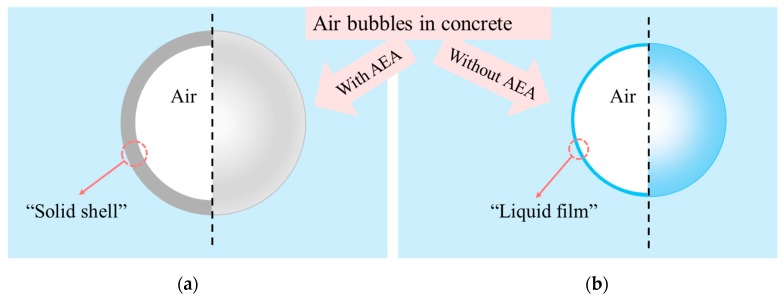
The “shell” and “film” on bubble surface (**a**) with air-entraining agent; (**b**) without air-entraining agent (adapted from Ley et al. [73]).

By using LTSEM, Corr et al. [41] found out that after cement hydration for 5 min, a special shell composed of irregular particles was formed around bubbles, and he pointed out that the shell might be the precipitate formed by the reaction of adsorbed anionic surfactant and calcium ion around bubbles. Ley et al. [73] separated and collected air bubbles from cement paste by tilting glass bottle, and found that the surface of air bubbles in air-entrained cement had a “shell”, as shown in Figure 6a. The shell had a self-healing ability that could withstand changes in external pressure. On the contrary, the surface of the bubble without air-entraining agent only had transparent or translucent “film”, which could easily be damaged, ruptured, and leaked, as shown in Figure 6b. It has been found out that the bubble “shell” is composed of small particles of different sizes from 100 nm to several μm, many of them are cement particles, including Ettringite rods [114].

#### 3.3.2. Superplasticizer

Water-reducing agents include ordinary water-reducing agents and high-efficiency water-reducing agents (that is, superplasticizers) [115]. Superplasticizers have a great influence on the stability of air-void [116,117]. Superplasticizers, such as calcium lignosulfonate (common superplasticizer) helps bubble formation, but the size of bubbles introduced is large and unstable [36]. Janusz found that superplasticizers could reduce surface tension, which in turn contributed to the generation of air bubbles and had an air-entraining effect [118]. Polycarboxylic acid-based high-performance water reducer can improve gas content ranging from 3% to 6% in concrete [44]. Adopting “elimination before introduction” and special air-entraining agent for polycarboxylic acid can stabilize bubbles and reduce bubble sizes [119]. Naphthalene-based superplasticizers increase bubble spacing coefficient and the loss of gas content.

From the latest research [120], a novel phosphate-modified polymer was synthesized, which showed great potential for practical application, and suggested that the polymeric surfactant with strong hydrophobic group improved foamability and foam stability.

#### 3.3.3. Salt Admixtures

Salt additives have an unavoidable effect on the air content of fresh air-entrained concrete [121]. When the effect of salts on electrostatic repulsion is greater than that on the hydrophobic association, the viscosity of solution decreases; when the effect of salts on the hydrophobic association is greater than the effect on electrostatic, the viscosity of the solution increases [112,120]. For some anionic air-entraining agents, after adding calcium salts, sedimentation occurs, and insoluble calcium soap is formed, which will reduce the effect of air-entraining agents. However, the precipitation will be adsorbed on the bubble film, increasing the mechanical strength of the bubble film, and enhancing the stability of the bubble film. The precipitation of anionic air-entraining agent can be avoided by mixing salt admixture and anionic air-entraining agent separately. The addition of sodium salt electrolyte will weaken the repulsion force between hydrophilic ions, increase the adsorption capacity of surfactant molecules on the surface, arrange the surfactant molecules more closely, and increase the ability of air-entraining agent [94,122]. The foam stability measurements (the foam half-life, height, and initial bubble size) showed that the foam stability was enhanced by some polymer, when compared to pure sodium dodecyl sulfate (an anionic surfactant) solutions [76].

### 3.4. Mixing Process

The formation of an air bubble is complicated during the stirring process, which is accompanied by the generation of new bubbles and the collapse of old bubbles. Mixing method, mixing time, mixing rate, etc. affect the generation and stability of air bubbles greatly [75,99,123]. Under certain conditions (including materials, mixing ratio, and external temperature), paying attention to the stirring process is auspicious for better bubble stability.

#### 3.4.1. Mixing Method

Mixing methods have important influences on bubble stability in concrete [38]. According to the mixing principle, mixing methods can be divided into self-falling mixing, forced mixing and vibration mixing, etc. [124,125]. Self-falling or self-dropping mixing is a free-fall mixing method in which the mixing barrel rotates the material at an appropriate speed. Forced mixing means that the mixing drum is fixed and the materials are stirred by the rotation of the blades on the rotating shaft inside the drum. Vibration mixing makes the material to vibrate intensely by the shaker. On the one hand, the bigger internal bubbles are shattered into smaller bubbles and disperse evenly; on the other hand, under the action of vibration, the water film on the surface layer of fresh concrete mixture breaks, and the air pours into the fresh concrete easily [126]. Through the experimental study on forced mixing and vibration mixing, it is believed that the vibration mixing contributes to the formation and stability of bubbles and effectively improves the air content of air-entrained concrete [125,126,127,128]. Compared with horizontal-shaft vibration stirring, vertical-shaft vibration stirring can increase the air content of fresh concrete to 3.5%. At a certain range of vibration intensity, the gas content is proportional to the vibration intensity. On the contrary, the vibration intensity is too powerful when outside the range, so bubbles in the mixture tend to escape, which is not advantageous for the formation and stability of bubbles [125].

#### 3.4.2. Mixing Time

Mixing time also affects bubble stability [53]. The air content of fresh concrete increases at first, then decreases when mixing time is prolonged [54]. That is, the air content of concrete rises when mixing time is increased within a certain range of time [51]. The best mixing time of air-entrained concrete is 2 to 3 min or 3 to 5 min [52,55]. Moreover, when the mixing time is in range of 80 to 140 s, with the mixing time prolonged [99], both the air content and the slump of concrete increases first and then decreases. When mixing time is 120 s, the concrete has reached the best frost resistance; when mixing time is 100 s, the maximum slump has reached. In terms of hydraulic concrete, the mixing time should be determined by experiments to ensure the required air content.

Schiessl et al. [129,130] divided the concrete mixing process into three stages: the dispersion period; the optimal mixing period, and the over mixing period. There is an optimal mixing time for concrete with a certain mixing proportion [131]. As for super high-performance concrete, when mixing time is less than stabilization time, increasing mixing time can raise air content and improve fluidity; when mixing time is longer than stabilization time, the air content will decline due to over-mixing [132].

#### 3.4.3. Mixing Rate

The mixing rate refers to the maximum linear velocity at the end of the mixing blade. It is generally believed that the air content of fresh concrete increases with the increase of mixing speed. The more intense the agitation, the faster the air induction and the more the air content increases [132,133]. Nevertheless, when the mixing process is changed, the effect of mixing speed on the air content of fresh concrete differs. With the traditional mixing method, the air content of fresh concrete increases with the increase of the mixing rate. When the mixing rate exceeds 1.6 m/s, the air content decreases with the increase of mixing rate, and the air content is within the range of 1.0% to 2.3%. With the secondary mixing process, the air content of fresh concrete decreases with the increase of the mixing rate. When the mixing rate exceeds 1.6 m/s, and when the air content is within the range of 1.1% to 1.75%, the air content of fresh concrete increases with the acceleration of the mixing rate [132].

Furthermore, under the action of vibration stirring, when the linear velocity of mixing is lower, the gas content of concrete is higher, and the gas content of fresh concrete gradually decreases as mixing speed increases [125].

### 3.5. Transportation, Pumping and Vibration

The loss of air content of fresh concrete during transportation [133], pumping [134] and vibration [55] is considerable. When the speed of concrete transporter is less than 5.5 r/min, the loss of gas content was minor, however, when it exceeds 5.5 r/min, the loss of gas content increases [34]. The longer the transport distance, the greater the gas content loss [33]. With the increase of rotation speed, the disturbance to concrete increases, the cement paste in concrete is continuously turned towards the surface of concrete, leading to the air bubbles in the paste to escape, and the loss of air content increases linearly with transportation time [133]. Therefore, long-time transportation is extremely detrimental to bubble stability. Additionally, the effect of pumping has a non-ignorable influence on bubble stability [134]. The loss of air content caused by pumping is approximately 1% to 1.5% [135].

Vibration frequency is significant to make fresh concrete consolidate. If the vibration frequency is too low, the concrete can’t be consolidated well; if the frequency is too high, the stability of the air bubble entrained is greatly damaged [136]. Long-time vibration and high-frequency vibration both cause significant loss of air content [33,55,137,138]. During the process of high-frequency vibration, concrete mixture liquefies. The solid particles tend to move to the most stable position due to gravity so that the internal voids become smaller. Most of the air entrapped in the mixture is discharged during the stirring process, and some of the air bubbles entrained by air-entraining agents also escape from the surface of the mixture, making the structure of concrete denser. Extending the high-frequency vibration time, a large number of small bubbles merge into large bubbles, the loss of gas content increases, the spacing coefficient of bubble and average bubble radius also increases [138]. The larger the water-cement ratio, the smaller the viscosity of concrete, and the greater the tendency of air bubbles to merge under high-frequency vibration.

Through simulation tests at standard atmospheric pressure and low atmospheric pressure, when vibrated within the range of 0 to 30 s, the air content of air-entrained concrete continued to decrease with the increase of vibration time, and the rate of reduction was large; when the time exceeded 30 s, the change of air content tends to be moderate. The spacing coefficient of air bubble and specific surface area of concrete both increase with the increase of vibration time [40]. Under high-frequency vibration, the concrete aggregate and cement paste are redistributed. The aggregates sink, the cement paste raises upward, and micro bubbles are carried out. The longer the vibration time, the greater the loss of air content [34,54]. Therefore, long time high frequency vibration will deteriorate the frost resistance [139]. During the construction process of concrete, the vibration time should be controlled reasonably. With the increase of high-frequency vibration time, the specific surface area of bubbles in hardened concrete increases, the average bubble diameter decreases, the total number of bubbles and the number of small bubbles reaches their peak and then rapidly decreases [42]. The order of damaging the foaming ability is: high frequency vibrating > vibrating rod > vibrating table > manual compacting. Adopting high-frequency vibrating and vibrating rod could reduce air content rapidly, therefore, the operating time of vibrating rods should be within 20 s [55].

### 3.6. Environmental Factors

#### 3.6.1. Temperature

The effect of temperature on the stability of air bubbles in fresh concrete relies on the properties of surfactants and the viscosity of the paste. The influence of temperature on surfactants depends on the nature of surfactants [15]. If the solubility of surfactants varies greatly with temperature, the air-entraining performance of surfactants increases with the rising of solubility [15,71,112]. Bubble stability changes in the same directions as air-entraining performance.

Powers [62] pointed out that the air content of air-entrained concrete decreases when the temperature increases. Generally, with a certain dosage of air-entraining agent, the gas content of concrete is higher at low temperature, and lower at high temperature [36]. When the temperature increases from 21 °C to 38.8 °C, the air content drops by 25%; contrarily, when the temperature decreases from 21 °C to 4.8 °C, the air content increases by 40% [140].

The temperature effect could be explained by the following mechanisms of acceleration of cement hydration [15]. The hydration of cement particles will speed up if the temperature is higher, so that more hydration products will be produced [141]. On the one hand, the viscosity of concrete mixtures will increase, which will be an energy for the formation of air bubbles, so that the air content of fresh concrete will decrease; On the other hand, with more AEA molecules adsorbed by the hydration products, the bubble stability will be affected.

#### 3.6.2. Atmospheric Pressure

**Figure 7:**
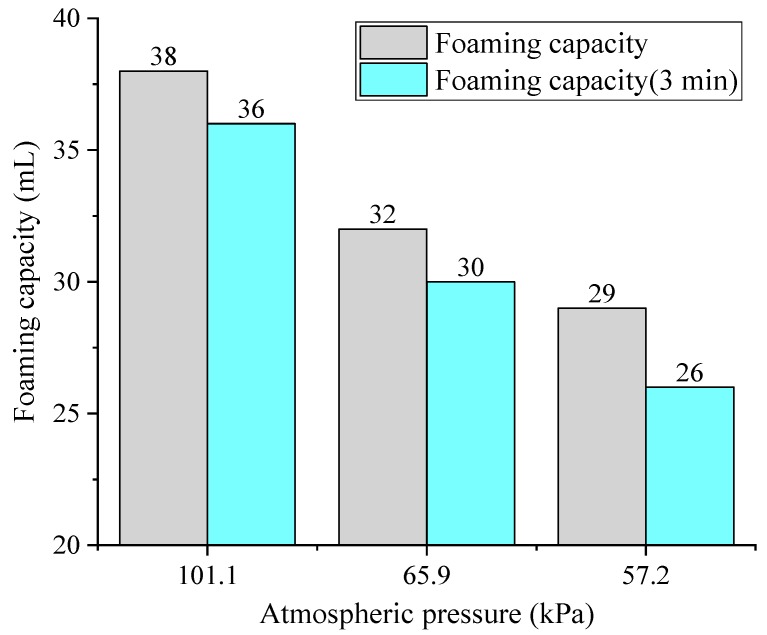
Foaming property of air-entraining agent (AEA) under different atmospheric pressure (data from Shi et al. [143]).

Presently, there is a consensus that low atmospheric pressure can significantly reduce the air-entraining ability of air-entraining agent [142]. The effect of atmospheric pressure on the performance of air-entraining agent was investigated at different atmospheric pressures. The result showed that when atmospheric pressure was lowered, the surface tension increased and the foaming capacity of air-entraining agent reduced, as shown in Figure 7 [143]. The air content in concrete linearly decreased with the decrease of atmospheric pressure when other conditions are constant [142,144,145].

**Figure 8:**
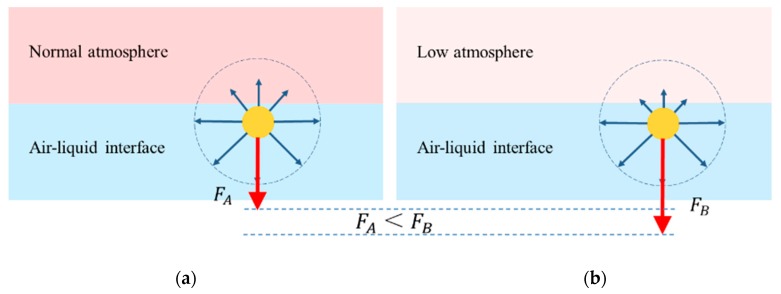
A molecule from interface under different atmospheric pressure (**a**) normal atmospheric pressure; (**b**) low atmospheric pressure.

Molecules at the air–liquid interface layer are much more affected by molecules in the liquid phase than molecules in the gas phase. Therefore, the molecules at air–liquid interface tend to be pulled into the liquid phase, leading the surface to shrink to a minimum. When atmospheric pressure decreases, the density of air also drops, thus the interaction between air and interface molecules decreases. Nevertheless, the density of the liquid is hardly affected by atmospheric pressure. So that a molecule of air–liquid interface under low atmospheric pressure is subjected to resultant force *F_B_* acting towards the interior direction of the liquid, and *F_B_* is much bigger than the resultant force *F_A_* under normal atmospheric pressure, as depicted in Figure 8. That is, the molecules on the surface are more unevenly stressed.

So that the surface tension of liquid increases under low atmospheric pressure [144]. The lower the atmospheric pressure, the higher the surface tension [146]. At high altitudes, the surface tension of water increases and the stability of air bubbles decreases, this makes it difficult to entrain air in concrete at high altitudes, so that the air content drops. Many efforts have been made to explore this phenomenon [147,148,149,150].

By using the low-pressure experimental apparatus, the low atmospheric pressure environment of the plateau area was simulated, and the influence of the decrease of atmospheric pressure on air content and bubble stability of air-entrained concrete was studied [40,148,149,150,151]. The research results indicated that the air content of air-entrained concrete decreased, the average diameter of air bubble increased, and the stability of the air bubble had been weakened significantly [152]. After that, the foaming property experiment of cement paste solution was conducted in Beijing and Tibet to investigate the air-entraining ability of air-entraining agents under different atmospheric pressure. The results showed that a decrease in atmospheric pressure could lead to a decrease in the air-entraining capacity of air-entraining agents [153]. Accordingly, by field experiments in high altitude area, it was concluded that the lower value of atmospheric made it difficult for bubbles to generate, and easy for bubbles to escape from the fresh concrete. As a result, bubble stability was deteriorated severely [154].

Nevertheless, the foaming stability of the air-entraining agent solution has been explored again experimentally by simulating a low atmospheric pressure environment recently [155,156]. Much surprisingly, an opposite conclusion was reached. The experimental results showed that the height of foam at low atmospheric pressure of 80 kPa and 60 kPa were nearly the same as that at normal pressure. Consequently, the reason for the decrease of gas content at low atmospheric pressure is not the deterioration of foaming performance of the air entraining agent solution, but other undiscovered reasons, which deserve further research.

## 4. Measures to Improve the Stability of Air Bubbles in Fresh Concrete

Nano-particles can promote interfacial interactions [157] and increase bubble stability [158]. Besides, the use of nano-particles could accelerate cement hydration, improve the mechanical properties of concrete [81], as well as enhance the resistance to water penetration [159]. The application of nano-particles in cement-based material is a current trend [158,160,161]. According to the mechanisms of bubble formation, bubble collapse and bubble stability as reviewed above, Nano-silica stabilized bubble could be the optimum choice to increase the formation and stability of bubbles and decrease the collapse of bubbles. Nanoparticle-stabilized bubbles are bubbles with nanoparticles adsorbed on their surface. There are three requirements for solid particle-stabilized bubbles [75]:

Solid particles should be much smaller than air bubbles. If solid particles are too big, they will sink into the liquid due to the gravity. Therefore, the solid particles should be much smaller than the air bubbles.

**Figure 9:**
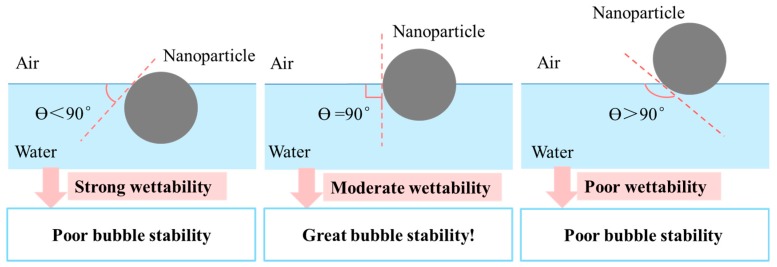
Bubble stability at different wetting angles.

The wettability of solid particles should be moderate. When solid particles have a strong wettability, that is the wetting angle Ө < 90°, the solid particles are easily lost with the drainage of liquid, thus they will have a little effect to stabilize bubble; when solid particles have a poor wettability, that is the wetting angle Ө > 90°, the interfacial membrane is too thin to stabilize bubble. When particles like nanoparticles have medium wettability, the wetting angle at the air–liquid interface is 90°, the surface elasticity of adsorbed layer of particle and the foaming stability reach the maximum value, as shown in Figure 9 [110].

There should be a high concentration of solid particles. When solid particles have met the former two requirements for the stability of the solid particle adsorption layer, if the concentration of solid particles is high, excess solid particles will accumulate in the liquid film between bubbles. When bubbles are close to each other, the nano-particles will hinder the drainage of the membrane and the coalescence of bubbles. In this way, the stability of the bubbles will be improved.

The nanoparticle-stabilized bubbles perform excellent stability [162,163,164,165,166,167,168]. When the mass fraction of nanoparticle reaches 1.5%, the foam stability increases by more than 6 times when compared to the control group, although the foaming capacity of the nanoparticle is 35% lower than that of the control group [163]. Nano-silica adsorbed on the surface of the air bubble can increase the viscosity of the bubble shell and inhibit the coalescence of air bubbles [169]. Yekeen et al. [170] found out that adding SiO_2_ nanoparticles into the liquid film of surfactant-contained foam improved the foam dynamic stability. Nano-silica can increase the thickness of the liquid film, which greatly enhanced bubble stability and decreased the bubble collapse. Meanwhile, Petit fabricated a single stable cement bubble by bubbling in a pool filled with micrometric silica particles [171].

Adding Nano-Silica into concrete can increase the viscosity of paste and the strength of the bubble film [172]. Nano-silica can effectively promote the elasticity of liquid film, thus preventing air bubbles from merging and the liquid film from breaking. Moreover, nano-silica can block Plateau channels, and enhance viscous resistance, thereby reducing the speed of bubble drainage, so that significantly raise the stability of bubbles. At the same time, it makes bubble size finer and bubble distribution uniform.

Furthermore, nano-silica can react with calcium hydroxide to form C-S-H on the bubble wall, which makes the wall of bubbles more compact. Nano-particles enlarge the surface area of air bubbles in contact with cement paste, and increase the frictional resistance of air bubbles when moving in the paste. So that the air bubbles maintain stable in fresh cement paste, which greatly improves the stability of air bubbles. Moreover, the use of nan- silica can increase the compressive and tensile strength of concrete [173,174]. Consequently, nano-silica can be applied to promote the stability of bubbles and the mechanical properties of concrete. The summary of previous research completed on bubble stability and nano-particles is shown in Table 2.

## 5. Conclusions

Fresh concrete is a complicated system and the stability of the air bubble is affected by many factors. In this work, the process of formation and collapse of air bubbles in concrete is characterized; the recent advances of major influencing factors of bubble stability are summarized; and the use of nano-silica is proposed to improve the stability of air bubbles in concrete.

The research on interfacial properties of bubble film in concrete is inadequate and poorly quantified. Most of the research on bubble stability in fresh concrete concentrate on field application and macroscopic phenomena, the quantified and microscopic research are rare. Furthermore, the quantified investigation of bubble stability reported focuses on the stability of the foam. However, the mechanisms of foam stability cannot be completely applicable to the bubble stability in fresh concrete.

There is not a consensus on the mechanisms of bubble stability in concrete. Surface tension exerts a great impact on bubble stability by reducing surface free energy and Plateau drainage, as well as increasing Gibbs surface elasticity. However, surface tension may not the only determinant of bubble stability. The stability of air bubbles in concrete may also be dominated by the strength of the bubble membrane and the gas diffusion rate through bubble film.

Since the application of nano-silica can increase the strength of bubble film, it has great potential in ameliorating the stability of air bubbles in concrete. The results showed that when the mass fraction of nano-particle reached 1.5%, the foam stability increased by more than six times when compared to the control group. However, the stability mechanisms of nano-particles stabilized bubble are based on the condition that the nano-particles are at a stationary state, without considering the effect of liquid drainage. Therefore, the mechanisms of nano-particles stabilized bubbles in concrete require further exploration.

In future research, factors that affect the stability of air bubbles in fresh concrete should be quantitatively and microscopically investigated. It is expected that this paper could provide some help for a sound understanding of bubble stability in concrete.

## Figures and Tables

**Table 1:** Summary of previous research completed on bubble stability and the strength of the liquid film.

Research	Experiment Apparatus/METHOD	Primary Conclusions	Summary
Ding et al. [98]	KSV Langmuir mini-trough	The monolayer strength of the air-entraining agent at gas–liquid interface determined the bubble stability directly. The higher the membrane strength, the more stable the air bubbles entrained by AEA, and the greater durability of concrete.	1. Surface viscosity and elasticity of bubble film greatly influence the stability of air bubbles. 2. Higher surface viscosity and elasticity could inhibit the liquid drainage of bubble film, which promotes the strength of bubble film.
Wang et al. [94]	A literature review	Although different scales of foam structure like the gas-water interface and the liquid film have been explored to clarify the mechanisms that control the foam stability, many questions remain unanswered yet.	
Yang [74]	Numerical simulation	The surface viscosity is an important factor affecting the process of liquid film drainage. When the surface viscosity is not considered, the surface of the liquid film presents a “flow” mode; when it is considered, it presents a “rigid” mode. With the increase of the surface viscosity, the drainage rate slows down obviously.	
Zhang [75]	Interfacial relaxation of tension experiment	The surface viscoelastic properties of nonionic surfactants NP-8, NP-10 and NP-12 were studied. The system with higher surface elasticity has higher bubble stability. The increase of the surface viscosity of the liquid film inhibits the thinning of the surface film.	
Naire et al. [104]	A mathematical model; experiment	The evolution of a vertically oriented thin liquid film drainage under gravity was studied. The results showed that increasing surface viscosity and the Marangoni effect could retard drainage, and consequently enhance film stability.	
Naire et al. [106]	A mathematical model	It was verified that in the limit of large surface viscosity and the Marangoni effect, the evolution of the free surface is that of a rigid film. Stable aqueous films can be formed in the regime of high surfactant concentrations.	
Saulnier et al. [108]	Film rupture experiments.	The results showed that for surfactants with high surface elastic modulus, the rupture began by the expansion of a thinning zone at the top of the film. The lifetime of films with small surface elastic modulus was much shorter than the ones with rigid interfaces.	

**Table 2:** Summary of previous research completed on bubble stability and nano-particles.

Research	Testing Apparatus	Primary Conclusions	Summary
Zang et al. [110]	Langmuir trough; Angle Microscope; Rheometer;	Foams prepared with nano-particles possessing intermediate hydrophobicity (i.e., the largest adsorption energy) were the most stable.	SiO_2_ nano-particles had great effects on bubble stability: 1. promoted the strength of liquid film; 2. increased surface viscosity and elasticity; 3. retarded gas diffusion and liquid drainage of bubble film; 4. prevent coalescence of bubbles.
Sun et al. [162,163]	Tracker Interfacial Rheometer	SiO_2_ nano-particles promoted mechanical strength of the liquid film, reduced the drainage, disproportionation and collapse rate of the bubble; the stability and surface dilatational viscoelasticity of foam increased with the increase of mass fraction of SiO_2_ nano-particles.	
Yang et al. [164]	A literature review	Nano-particles (E.g. Nano silica) adsorbed at interface could increase the surface elasticity of bubble; block the flow of liquid in the film; delay the thinning of film, and prevent the coalescence of bubble. In addition, the foam produced when contact angle is around 90° was stable.	
Li et al. [165,166,167]	Tracker interfacial rheometer; viscometer; centrifuge; microscope	Nano-particles could attenuate the drainage of the liquid membrane and reduce the coalescence of bubbles, which played a critical role in protecting bubbles.	
Lu et al. [168]	Interface rheometer, microscope; viscosity meter; gas diffusion testing device	The SiO_2_ nano-particles foam showed excellent resistance to liquid drainage and bubble coalescence. Besides, the strength of the bubble liquid film is relatively high.	
She et al. [169]	Viscometer XCT Precision system (YXLON, Germany)	Nano-silica could slow the coalescence and disproportionation of bubbles and increase the viscosity of the bubble wall, thus preventing gas transfer and drainage between gaseous and liquid phases.	
Yekeen et al. [170]	Leica EZ4 HD stereo microscope	The presence of SiO_2_ nano-particles in the surfactant solution improved the foam dynamic stability in water-wet and oil-wet porous media.	
Petit et al. [171]	SEM; A device to fabricate stable and fully covered solid cement bubble	Bubble stability is shown to be governed mainly by particle covering rate, which is maximized when the particle wetting angle prior to liquid approaches π/2.	
Du [172]	SEM, viscometer Optical microscope	Nano silica had great effects on bubble stability. It enhanced the viscosity of the solution and the strength of film; blocked Plateau channels and nodes, Moreover, bubble size was refined.	
